# Five-Year Outcome after Coronary Artery Bypass Surgery in Survivors of Out-of-Hospital Cardiac Arrest

**DOI:** 10.3389/fsurg.2015.00002

**Published:** 2015-01-21

**Authors:** Matti-Aleksi Mosorin, Maté Lantos, Tatu Juvonen, Fausto Biancari

**Affiliations:** ^1^Department of Surgery, Oulu University Hospital, Oulu, Finland

**Keywords:** out-of-hospital cardiac arrest, cardiac arrest, coronary artery bypass surgery, myocardial revascularization, myocardial infarction

## Abstract

**Objective:** The aim of this study was to evaluate the role of coronary artery bypass grafting (CABG) in patients with out-of-hospital cardiac arrest (OHCA).

**Methods:** The immediate and 5-year outcome after CABG of a consecutive series of 48 patients who survived OHCA was compared with those of control patients having had a recent myocardial infarction without ventricular arrhythmias.

**Results:** All OHCA patients were found to have suffered myocardial infarction-related cardiac arrest. The mean delay from OHCA to CABG was 10.3 ± 13.0 days. Despite not statistically significant, the risk of 30-day postoperative mortality was higher among OHCA patients than control patients (6.3 vs. 0%, *p* = 0.24, propensity score adjusted analysis: *p* = 1.00). Cardioverter defibrillator was implanted in two patients who were alive 3.8 and 4.4 years after CABG, respectively. At 5-year, the overall survival rate was 80.7% in OHCA patients and 84.5% in control patients (*p* = 0.98, propensity score adjusted analysis: *p* = 0.87), and survival freedom from fatal cardiac event was 86.1% in OHCA patients and 86.5% in control patients (*p* = 0.61; propensity score adjusted analysis: *p* = 0.90).

**Conclusions:** Early and 5-year survival rates after CABG in OHCA patients are excellent even when cardioverter defibrillator is very selectively implanted. The early and intermediate results CABG suggest a confident approach toward surgical revascularization in this critically ill patient population.

## Introduction

The role of coronary revascularization strategies in patients with out-of-hospital cardiac arrest (OHCA) is not well established. Heterogeneity in the definition, ascertainment, etiology, and treatment of OHCA varies greatly among studies and prevents conclusive results on the best treatment strategy (1). There are a number of recent prospective studies, which evaluated the outcome of OHCA patients with associated coronary artery disease after percutaneous coronary intervention reporting immediate postoperative mortality rates ranging from 19 to 51% (1–3). On the contrary, data on the immediate and late outcome of these patients after coronary artery bypass grafting (CABG) are scarce (4–6).

The purpose of this study was to investigate the prognosis of patients with OHCA who underwent CABG during the last decade and to compare their outcome with those of matched controls.

## Materials and Methods

This retrospective study included a series of consecutive of patients who survived a recent OHCA and who underwent CABG at the Department of Surgery, Oulu University Hospital, Oulu, Finland, from January 2000 to December 2009. Permission to perform this study was not asked at the time of data collection as our Institutional Review Board did not require any permission for retrospective collection of patients’ data. A control series of patients with recent myocardial infarction (<3 months prior to CABG) has been randomly chosen from our database and matched in a 1:1 fashion for age (±1 year), gender and date (±1 month) of operation.

Study design and data retrieval were planned according to the Newcastle–Ottawa scale criteria (http://www.ohri.ca/programs/clinical_epidemiology/oxford.htm) regarding representativeness of the exposed cohort (consecutive series representative of survivors of OHCA without any exclusion criteria), selection of the non-exposed cohort (random, matched sample of patients without any other exclusion criteria than recent myocardial infarction, same age, same gender, and operation carried out during the same period), ascertainment of exposure (data retrieval from electronic records), demonstration that outcome of interest was not present at start of the study, comparability of cohorts (cohorts matched for more than two important factors), assessment of outcome (blind assessment of the outcome), follow-up length (a 5-year period is likely enough to detect any difference in outcome), and adequacy of follow-up (follow-up data retrieved from an official national registry).

These patients were identified from our institutional database and data were reviewed to collect variables of interest. Patients who suffered of in-hospital cardiac arrest were excluded from this analysis. Follow-up data were retrieved from our institutional records for those patients residing in our catchment area, otherwise from central hospitals’ records for those residing in other catchment areas. Clinical variables were defined according to the EuroSCORE criteria (7). Operative risk was estimated by the EuroSCORE (7) and its modified version (8, 9). Cause and date of death were retrieved on January 2010 from the Finnish National Registry, Statistics Finland – Tilastokeskus. This implies that in this study there were no lost to follow-up or any patients with incomplete data on cause of death.

### Statistical analysis

Statistical analysis was performed using a SPSS statistical software (SPSS v. 15.0.1, SPSS Inc., Chicago, IL, USA). Continuous variables are reported as the mean ± SD. The Fisher exact’ test and the Mann–Whitney tests were used for univariate analysis. Intermediate outcome was assessed by the Kaplan–Meier (log-rank test) and Cox proportional hazards methods. Non-parsimonious logistic regression was performed to estimate a propensity score and to assess the risk of assignment to these study groups, i.e., OHCA vs. control group. Receiver operating characteristics (ROC) analysis was performed to assess the predictive ability of this regression model. The estimated propensity score was employed for adjusted analyses evaluating the immediate and late outcome of these patients. A *p* < 0.05 was considered statistically significant.

## Results

Clinical characteristics and operative data are summarized in Table 1. No significant differences were observed between the OHCA and control groups in terms of baseline variables, but the former group had significantly higher EuroSCORE and modified EuroSCORE, mainly because of these patients’ critical preoperative status related to OHCA event as defined by these two risk scoring methods. A propensity score was estimated and had an area under the ROC curve of 0.757 (95%CI 0.662–0.853).

**Table 1:** **Clinical and operative data on patients with out-of-hospital cardiac arrest and matched controls with recent myocardial infarction <3 months who underwent isolated coronary artery bypass surgery**.

	Out-of-hospital cardiac arrest (48 patients)	Controls (48 patients)	*p*-value
Age (years)	65.2 ± 1.2	65.2 ± 1.2	0.97
Females	6 (12.5)	6 (12.5)	1.00
Dyslipidemia	16 (33.3)	18 (37.5)	0.67
Pulmonary disease	6 (12.5)	4 (8.3)	0.74
Hypertension	25 (52.1)	16 (33.3)	0.06
Serum creatinine (mmol/l)	89 ± 5	83 ± 3	0.78
Atrial fibrillation	9 (18.8)	7 (14.6)	0.79
Transient ischemic attack	1 (2.1)	4 (8.3)	0.35
Stroke	3 (6.3)	0 (0)	0.24
Neurological dysfunction	4 (8.3)	1 (2.1)	0.36
Extracardiac arteriopathy	4 (8.3)	3 (6.3)	1.00
Previous vascular/endovascular surgery	2 (4.2)	2 (4.2)	1.00
Previous cardiac surgery	1 (2.1)	0 (0)	1.00
Previous percutaneous transluminal angioplasty	4 (8.3)	1 (2.1)	0.36
Left main stenosis >50%	20 (41.7)	15 (31.3)	0.29
LVEF >50%	26 (54.2)	30 (62.5)	0.70
30–50%	18 (37.5)	15 (31.3)	
<30%	4 (8.3)	3 (6.3)	
Critical preoperative status	32 (66.7)	2 (4.2)	<0.0001
Preoperative inotropic support	7 (14.6)	4 (8.3)	0.52
Tracheal intubation at OR arrival	8 (16.7)	2 (4.2)	0.09
Nitrates infusion at operating room arrival	16 (33.3)	18 (37.5)	0.67
Systolic pulmonary a. pressure >60 mmHg	2 (4.2)	1 (2.1)	1.00
Cardiac index (l/min/m^2^)	2.7 ± 0.6	2.6 ± 0.7	0.47
Emergency operation	6 (12.5)	5 (10.4)	0.86
Off-pump surgery	20 (41.7)	23 (47.9)	0.54
Diseased ascending aorta	5 (10.4)	5 (10.4)	1.00
At least one mammary a. graft	46 (95.8)	46 (95.8)	1.00
No. distal anastomoses	3.7 ± 0.1	3.8 ± 0.1	0.84
Logistic EuroSCORE (%)	14.3 ± 2.3	7.8 ± 1.6	0.003
Logistic modified EuroSCORE (%)	5.5 ± 1.1	1.1 ± 0.4	<0.0001

*Continuous variable are reported mean ± SD; LVEF, left ventricular ejection fraction; OR, operating room*.

*Variables definition criteria are according to EuroSCORE. Values in parentheses are percentages*.

Before resuscitation, 44 patients had ventricular fibrillation (91.7%), 1 had asystole (2.1%), 1 had pulseless electrical activity (2.1%), whereas in 2 patients there was no clear information about the type of arrhythmia at resuscitation. All these patients were found to have suffered myocardial infarction-related cardiac arrest. Accordingly, myocardial enzymes were found to be elevated in all OHCA patients, but they were not taken into account in this analysis because the heterogeneity of types and method of measurement of these enzymes.

Hypothermia treatment after successful resuscitation was employed in 14 OHCA patients (29.2%) because of neurological derangement related with cardiac arrest. Hypothermia was carried out at about 32.0–33.0°C for 24 h. Four of these patients had neurological deficit even after hypothermia treatment. Neurological status could not be assessed in eight patients who were intubated at arrival in operating room because of their unstable cardiopulmonary conditions. However, in all the latter patients, attempts to relieve sedation preoperatively revealed severe neuropsychological derangements.

At coronary angiography, among OHCA patients, 41 (85.4%) had 3 vessel disease, 6 (12.5%) had 2 vessel disease and 1 (2.1%) had 1 vessel disease. Three OHCA patients (6.3%) underwent surgical revascularization within 30 days after percutaneous coronary intervention. The mean delay from OHCA to CABG was 10.3 ± 13.0 days (median 7.0 days, interquartile range 7.5) and only two patients (4.2%) were operated on elective basis. Immediate postoperative outcome is summarized in Table 2. Despite not statistically significant, 30-day postoperative mortality was higher among OHCA patients than controls (6.3 vs. 0%, *p* = 0.24, propensity score adjusted analysis: *p* = 1.00). As expected on the basis of the preoperative status, neuropsychological derangements were more frequently observed among OHCA patients (27.1 vs. 8.3%, *p* < 0.0001; propensity score adjusted analysis: *p* = 0.14). Postoperative stroke occurred in one patient in each study group.

**Table 2:** **Immediate and late outcome patients with out-of-hospital cardiac arrest and matched controls with recent myocardial infarction <3 months who underwent isolated coronary artery bypass surgery**.

	Out-of-hospital cardiac arrest (48 patients)	Controls (48 patients)	Unadjusted *p*-value	Propensity score adjusted *p*-value
30-day mortality	3 (6.3)	0 (0)	0.24	1.00
In-hospital mortality	2 (4.2)	0 (0)	0.50	1.00
Stroke	1 (2.1)	1 (2.1)	1.00	0.53
Neuropsychological derangement	13 (27.1)	4 (8.3)	0.03	0.14
Cardiac low-output syndrome^a^	5 (10.4)	6 (12.5)	1.00	0.96
Need of inotropes >12 h	10 (20.8)	13 (27.1)	0.47	0.29
Intra-aortic balloon pump	3 (6.3)	1 (2.1)	0.62	0.21
*De novo* dialysis	0 (0)	1 (2.1)	0.32	1.00
Resternotomy for bleeding	2 (4.2)	2 (4.2)	1.00	0.69
Red blood cells units transfused	2.3 ± 0.4	2.2 ± 0.4	0.06	0.22
Sepsis	2 (4.2)	1 (2.1)	1.00	0.81
Atrial fibrillation	21 (43.8)	20 (41.7)	0.84	0.90
Pneumonia	7 (14.6)	5 (10.4)	0.76	0.96
Length of stay in the ICU (days)	2.2 ± 0.3	3.0 ± 0.6	0.59	0.25

*^a^Postoperatively cardiac index <2.0 l/min/m^2^ at least twice; ICU, intensive care unit. Values in parentheses are percentages*.

Electrophysiological study was performed in four OHCA patients and failed to reveal any sustained ventricular tachycardia. Cardioverter defibrillator was implanted in two other patients in whom otherwise the electrophysiological study was not performed. These two patients were alive 3.8 and 4.4 years after CABG, respectively.

The mean length of follow-up was 4.9 ± 0.4 years for OHCA patients and 4.6 ± 0.4 years for control patients. Cause of death was retrieved in all cases, but in one patient who has recently died and his data was not yet achieved by the Finnish National Registry. The latter cause of death was thus categorized as undetermined.

**Figure 1:**
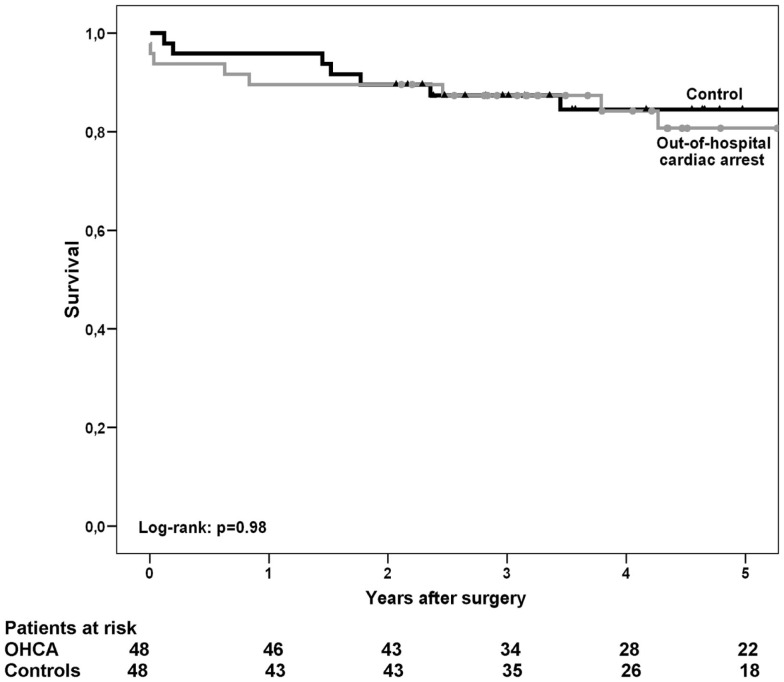
**Kaplan–Meier’s estimate of overall survival after coronary artery bypass grafting in patients with out-of-hospital cardiac arrest (OHCA) and in control patients who suffered myocardial infarction without ventricular arrhythmia within 3 months prior to surgery**.

Despite a slightly higher early mortality among the OHCA patients, at 5-year the overall survival rate was similar in the study group, having been 80.7% in OHCA patients and 84.5% in control patients (10 patients vs. 10 patients, respectively, log-rank test: *p* = 0.98, Figure 1). Propensity score adjusted analysis confirmed that no difference in survival existed between the study groups (*p* = 0.87, HR 1.09, 95%CI 0.39–3.05). At 5-year, survival freedom from fatal cardiac event was 86.1% in OHCA patients and 86.5% in control patients (6 patients vs. 8 patients, respectively, log-rank test: *p* = 0.61; propensity score adjusted analysis: *p* = 0.90, HR 0.93, 95%CI 0.28–3.08).

## Discussion

The present results confirm that 5-year survival after CABG among survivors of OHCA is about 80% or more as previously observed in three previous studies on this topic (4–6). Contrary to the study by Mangi and colleagues (6), the herein observed good results have been achieved despite cardioverter defibrillators were implanted very selectively. In fact, in the study by Mangi and colleagues (6), electrophysiological study was performed in 88% of patients and cardioverter defibrillator was implanted in 32% of patients. Furthermore, the mean delay between OHCA event and CABG was about 29 days in the study by Mangi et al. (6), whereas it was 10 days in the present study with 12.5% of patients requiring emergency operation. In the study by Every and colleagues (4), CABG was associated with an operative mortality of 2.3%, but again the mean delay from the OHCA and surgery was rather long (43 days), which may suggest a bias in referring these patients for surgical myocardial revascularization. Interestingly, only 40% of their patients were operated during the hospital stay after resuscitation (4). This may indicate a different treatment policy along with different patients’ conditions, which in turn may explain also our higher operative mortality as compared to previous studies (4, 6). The herein observed early mortality can be anyway considered acceptable in view of the high operative risk of OHCA patients. Interestingly, the observed in-hospital mortality was similar to the one predicted by the modified EuroSCORE risk scoring method (8, 9).

Coronary artery bypass grafting has been recognized as an effective treatment to reduce the risk of ventricular tachycardia (4, 10). Every and colleagues (4), in 1992, reported on a series of 85 patients who underwent CABG after OHCA and compared them with a series of OHCA patients treated medically. These patients did not undergo either percutaneous coronary intervention or implantation of cardioverter defibrillator. Acute myocardial infarction was detected in 17% of CABG patients and in 32% of medically treated patients. During a mean follow-up of 4.9 years, 13% of CABG treated and 42% of medically treated patients had a second cardiac arrest. Twenty-six percent of CABG patients and 62% of medically treated patients died or had a second non-fatal cardiac arrest. These findings are of clinical importance as none of their patients underwent percutaneous coronary intervention or received a cardioverter defibrillator; therefore, the investigators had the opportunity to evaluate the real impact of CABG-only treatment on the outcome of OHCA patients. Kelly and colleagues (5) reported that 80% of patients had inducible ventricular arrhythmias prior to CABG and 45% after CABG. Despite the high rate of postoperative inducible arrhythmias, they reported 5-year survival of 88%, a freedom from fatal cardiac events of 98%, and an arrhythmia-free survival of 88%.

This study was not planned to address the question whether CABG achieves better results than percutaneous coronary intervention. We believe that such a comparative analysis is not feasible neither in a randomized study nor in a propensity adjusted analysis as percutaneous coronary intervention is usually performed in these patients as a first treatment strategy in the urgent/emergency setting. In fact, it is likely that cardiologists face more frequently than cardiac surgeons with such critically ill patients, possibly with severe neurological complications after resuscitation, and are thus more prone to perform percutaneous coronary interventions as a compassionate and much less invasive alternative treatment to surgical revascularization. This may explain the high immediate mortality rates after percutaneous coronary intervention (1–3). Anyway, such good early and intermediate results observed after CABG suggest a confident approach toward surgical revascularization also in this critically ill patient population.

The retrospective nature is the main limitation of this study. However, in Finland invasive treatment of coronary artery disease is centralized and patients’ follow-up data can be reliably retrieved from our University hospital as well as from central hospitals from which patients are referred. We do not have data whether patients died of recurrent malignant arrhythmia, but survival data from our national registry provided reliable data about the cause of late death. The mean follow-up of this small series was about 5 years and we do not know whether it is long enough to detect any major differences in the outcome of these patients. The present study includes patients who suffered acute myocardial infarction-related OHCA and needed prompt coronary revascularization. This has likely eliminated a selection bias, which was otherwise possible in previous studies reporting of patients treated a mean of 30–43 days after OHCA, some of them not having suffered myocardial infarction (4, 5).

In conclusion, the present results suggest that isolated and complete surgical revascularization of the myocardium in patients with acute myocardial infarction-related OHCA can be associated with a good 5-year prognosis. Such good early and intermediate results observed after CABG suggest a confident approach toward surgical revascularization in this critically ill patient population.

## Conflict of Interest Statement

The authors declare that the research was conducted in the absence of any commercial or financial relationships that could be construed as a potential conflict of interest.
